# Simultaneous E-cadherin and PLEKHA7 expression negatively affects E-cadherin/EGFR mediated ovarian cancer cell growth

**DOI:** 10.1186/s13046-018-0796-1

**Published:** 2018-07-11

**Authors:** Katia Rea, Francesca Roggiani, Loris De Cecco, Francesco Raspagliesi, Maria Luisa Carcangiu, Joyce Nair-Menon, Marina Bagnoli, Ileana Bortolomai, Delia Mezzanzanica, Silvana Canevari, Antonis Kourtidis, Panos Z. Anastasiadis, Antonella Tomassetti

**Affiliations:** 1 Unit of Molecular Therapies, Department of Research, Via Amadeo 42, 20133 Milan, Italy; 2 Genomics, Department of Applied Research and Technology Development, Via Amadeo 42, 20133 Milan, Italy; 3 Gynecology Oncology Unit, Department of Surgery, Via Amadeo 42, 20133 Milan, Italy; 4 Unit of Anatomic Pathology I, Deparment of Anatomic Pathology, Fondazione IRCCS Istituto Nazionale dei Tumori, Via Amadeo 42, 20133 Milan, Italy; 5 Department of Regenerative Medicine and Cell Biology, Medical University of South Carolina, 173 Ashley Avenue, Charleston, SC 29425 USA; 6 Present address: Telethon Institute for Gene Therapy (SR-TIGET), Division of Regenerative Medicine, Stem Cells and gene Therapy, IRCCS San Raffaele Scientific Institute, Via Olgettina 60, 20132 Milan, Italy; 7 Department of Cancer Biology, 4500 San Pablo Road, Jacksonville, FL 32224 USA; 8 Mayo Clinic Comprehensive Cancer Center, Mayo Clinic, 4500 San Pablo Road, Jacksonville, FL 32224 USA

**Keywords:** E-cadherin, Epithelial ovarian cancer, EGFR, CDK5, PLEKHA7

## Abstract

**Background:**

The disruption of E-cadherin-mediated adhesion is considered an important driver of tumor progression. Nevertheless, numerous studies have demonstrated that E-cadherin promotes growth- or invasion-related signaling, contrary to the prevailing notion. During tumor progression, epithelial ovarian cancer (EOC) maintains E-cadherin expression and can positively affect EOC cell growth by contributing to PI3K/AKT activation. In polarized epithelia PLEKHA7, a regulator of the zonula adherens integrity, impinges E-cadherin functionality, but its role in EOCs has been never studied.

**Methods:**

Ex-vivo EOC cells and cell lines were used to study E-cadherin contribution to growth and EGFR activation. The expression of the proteins involved was assessed by real time RT-PCR, immunohistochemistry and western blotting. Cells growth and drug susceptibility was monitored in different 3-dimensional (3D) systems. Recombinant lentivirus-mediated gene expression, western blotting, immunoprecipitation and confocal microscopy were applied to investigate the biological impact of PLEKHA7 on E-cadherin behaviour. The clinical impact of PLEKHA7 was determined in publicly available datasets.

**Results:**

We show that E-cadherin expression contributes to growth of EOC cells and forms a complex with EGFR thus positively affecting ligand-dependent EGFR/CDK5 signaling. Accordingly, 3D cultures of E-cadherin-expressing EOC cells are sensitive to the CDK5 inhibitor roscovitine combined with cisplatin. We determined that PLEKHA7 overexpression reduces the formation of E-cadherin-EGFR complex, EGFR activation and cell tumorigenicity. Clinically, PLEKHA7 mRNA is statistically decreased in high grade EOCs respect to low malignant potential and low grade EOCs and correlates with better EOC patient outcome.

**Conclusions:**

These data represent a significant step towards untangling the role of E-cadherin in EOCs by assessing its positive effects on EGFR/CDK5 signaling and its contribution to cell growth. Hence, the inhibition of this signaling using a CDK5 inhibitor exerts a synergistic effect with cisplatin prompting on the design of new therapeutic strategies to inhibit growth of EOC cells. We assessed for the first time in EOC cells that PLEKHA7 induces changes in the asset of E-cadherin-containing cell-cell contacts thus inhibiting E-cadherin/EGFR crosstalk and leading to a less aggressive tumor phenotype. Accordingly, PLEKHA7 levels are lower in high grade EOC patient tumors and EOC patients with better outcomes display higher PLEKHA7 levels.

**Electronic supplementary material:**

The online version of this article (10.1186/s13046-018-0796-1) contains supplementary material, which is available to authorized users.

## Background

Cadherins mediate cell-cell adhesion through a mechanism whereby proteins protruding from opposing cells and interacting with each other at the cellular adherens junctions (AJs). The epithelial-specific E-cadherin, together with the cytoplasmic proteins β-, p120, and α-catenins, is connected to the actin cytoskeleton thus helping to maintain epithelial integrity [1]. In cancer, the switch in expression from E- to N-cadherin is considered a key event in the cellular epithelial-to-mesenchymal transition (EMT) that takes place as neoplasm progresses and is associated to chemoresistance [2]. Conversely, in epithelial ovarian cancer (EOC) cells E-cadherin shows a high level of expression during tumor progression [3–6] being often expressed together with N-cadherin whose role in modulating signalling activation in these tumors is still unclear besides its association to chemoresistance [7]. More than 40% of high-grade (HG) EOC patients present two different populations of tumor cells at diagnosis, those belonging to the solid peritoneal masses, and those mainly present as multicellular aggregates (MCAs) in ascites [8]. The tendency to form these MCAs suggests that cell-cell adhesion mediated by cadherins exerts a pivotal role for the persistence of these aggregates in the peritoneum during EOC progression. We previously reported that the formation of cell-cell contacts through E-cadherin contributes to mechanisms of proliferation in EOC cells by recruiting the PI3K-p85 subunit to the cell membrane, thus leading to PI3K/AKT activation [4]. In polarized epithelia, the growth suppressor role of E-cadherin is dependent on the expression of PLEKHA7, a component of the zonula adherens (ZA) [9].

In HG-SOCs, the receptor tyrosine kinases (RTKs), expressed in the majority of these tumors, greatly contribute to the survival, proliferation, and invasion. EGFR gene is amplified, but not mutated, in up to 37% of samples analyzed [10]. EGFR can contribute to EOC aggressiveness by increasing proliferation, migration and invasion as well as resistance to platinum compound [11–15]. Nonetheless, therapeutic approaches targeting EGFR gave poor response in HG-SOCs [16]. One possible explanation for this is that EGFR expression/amplification may not directly correlate with EGFR activation and signaling in HG-SOCs. We indeed reported that EGFR is not always expressed on the membrane and therefore activated in EOCs [17]. Furthermore, we identified a subset of EOCs in which EGFR activation positively regulates inflammation-related pathways and drug sensitivity [17, 18]. We have also described a novel signaling pathway whereby EGF-activated EGFR leads to the activation of CDK5 and increased proliferation in EOC, thyroid carcinoma, and melanoma cells [19]. These observations further support a role for EGFR in the pathophysiology of EOC and argue that different EGFR-dependent signalings are crucial to identify HG-SOC patients who may benefit from target-guided therapies.

In both normal and malignant cell models, EGFR activation may be positively or negatively affected by E-cadherin expression and functionality [20–22], however, a functional link between the presence of E-cadherin on EOC cells and EGFR signaling activation has not been deeply investigated.

Herein, using cellular models and patient-derived EOC samples, we further assess the pro-tumorigenic function of E-cadherin by investigating i) the cross-talk between E-cadherin and EGFR; ii) the possible modulation of EGFR activation and signaling to the down-stream effector CDK5 by E-cadherin; iii) the role of PLEKHA7 expression in modulating E-cadherin behavior and EOC patient outcome.

## Methods

### Antibodies and reagents

The list of the primary and secondary Abs, as well as working dilution for each assay, is reported in Additional file 1: Table S1. Human recombinant EGF was from PeproTech (London UK). Lipofectamine2000 or Lipofectamine3000 were purchased from Invitrogen (Carlsbad, CA, USA).

### Cells and patient samples

The EOC cell lines used in this study were: OAW42, kindly provided by Dr. Ulrich (Dr. A Ullrich, Martinsried, Germany); SKOV3 from ATCC; OVCAR5 and OVCAR4, provided by Dr. Camalier (NCI-NIH, USA); NL3507, from Dr. van den Berg-Bakker (Leiden, the Netherlands) [23]; IGROV-1 kindly provided by Dr. Bénard (Paris, France) [24]. Caco2 colon epithelial cells (ATCC) were used as positive control for PLEKHA7 expression and localization [9]. Cells were maintained in RPMI 1640 medium or EMEM (for OAW42) (Sigma Aldrich, St. Louis, MO) supplemented with 10% FCS (Hyclone, Logan, UT), 2 mM L-glutamine, at 37 °C in a humidified atmosphere of 5% CO_2_. Cells were genotyped at the Functional Genomic facility of our Institute using a Stem Elite ID System (Promega, Madison, WI, USA), according to the manufacturer’s instructions and ATCC guidelines. Cells were routinely confirmed to be mycoplasma-free by a MycoAlert Mycoplasma Detection Kit (Lonza, Basel, Switzerland). Cells, grown at 70% confluency and starved for 24 h, were stimulated with 20 ng/ml EGF. For starvation, cells were grown in complete medium depleted of FCS.

Twenty ascites samples from HG-SOC patients were collected. The Institutional Review Board approved the use of archived material and ascites, as well as clinical data. All clinical specimens were accompanied by informed consent from all patients to use the excess biological material for investigative purposes. The histological selection of patients was based on an advanced stage at diagnosis and the presence of ascites at surgery. Cytological analysis confirmed that the ascites mainly contained tumor cells as also demonstrated by the expression of the epithelial marker claudin-4, evaluated by western blotting. Cells from HG-SOC ascites were collected by centrifugation and the suspension of collected cells was seeded in a flask for 30 min to allow immune cells to adhere to the plastic. Non-adherent cells were recovered by centrifugation and processed for further analysis. For samples #9, #11, and #13–18, both solid FFPE or frozen biopsies and MCAs from ascites were analyzed by RT-PCR and IHC. IHC analyses with anti-E-cadherin and -PLEKHA7 Abs were performed on these samples.

Two patient-derived xenografts (PDXs), arising from serial intra-peritoneal (i.p.) xenotransplantion in SCID mice of cells from HG-SOC ascites, collected at the time of the primary surgery, were used. PDX#19 was from a 47-year-old patient and PDX#20 was from a 61-years-old patient. Cells from HG-SOC sample #21 were transiently transfected with a siRNA for E-cadherin, as described below, starved for 24 h and stimulated with 20 ng/ml EGF overnight in FCS depleted medium. Total cell lysates were prepared from EGF stimulated cells, which were still viable.

### RNA extraction and real-time RT–PCR analysis

Total RNA extraction and real-time RT-PCR were performed as described [25].

### Immunohistochemistry (IHC)

IHC with anti-E-cadherin Ab was performed as described [4] on the human HG-SOC samples reported above. Positivity or negativity of staining was assessed independently by two observers (MLC and AT). For PLEKHA7, antigen retrieval was carried out at 95 °C for 10 min in citrate buffer (pH 6).

### Western blotting and Immunoprecipitation (IP)

Cells were washed with ice-cold PBS containing Na_3_VO_4_ (0.1 mM) and lysed with NuPAGE® LDS sample buffer (1X) (Invitrogen) under reducing conditions. Western blotting and IP were performed as described [4]. The lysis buffer used for IPs with anti-E-cadherin and -EGFR contained 1,1% octyl-β-glucoside [4] while for IP with anti-PLEKHA7 contained 1% IGEPAL CA-630 (Sigma-Aldrich). Quantization on western blotting was assessed by using ImageJ software.

### 3D culture and cell viability assay

3D cultures were performed growing cells on an Algimatrix™ (ThermoFisher Scientific, Waltham, MA, USA) scaffold or in growth factor-reduced Matrigel® (BD Biosciences, Bedford, MA), essentially as described [25]. E-cadherin-silenced or control-transfected cells (1 × 10^3^) were suspended in Matrigel® and then seeded in 48-well culture plates. Plates were first incubated for 30 min at 37 °C and then complete medium was added. To evaluate cell viability of MCAs grown in Algimatrix™ (ThermoFisher Scientific) scaffolds, mitochondrial activity was measured with the CellTiter-Glo® Luminescent Cell Viability Assay performed according to the manufacturer’s instructions (Promega, Madison, WI). Cell viability of MCAs grown in Algimatrix™ was also evaluated with LIVE/DEAD™ Viability/Cytotoxicity Kit for mammalian cells performed according to the manufacturer’s instructions (ThermoFisher Scientific).

### siRNA and LZRS plasmid transient transfection

Cells were transfected with 40 pmol/ml siRNA specific for E-cadherin or EGFR or non-silenced siRNA as negative control (Quiagen-Xeragon, Germantown, MD). siRNA specific for EGFR was purshed from Dharmacon (ThermoFisher Scientific). For transient PLEKHA7 overexpression, OAW42 cells were transfected with the previously described LZRS-PLEKHA7-Myc vector [9]. Transfection was performed using Lipofectamine 2000 according to the manufacturer’s protocol. Whole cell lysates were prepared 48 h after transfection. Alternatively, stable PLEKHA7-expressing cells were obtained by retroviral infection as described [9].

### Immunofluorescence (IF) and confocal microscopy

EOC MCAs, released from the scaffold as described above, as well as cells grown adherent on 8-well glass chamber slides (Nalge Nunc International NY, USA), were fixed with 2% paraformaldehyde for 20 min and permeabilized for 10 min in PBS containing 0.1% Tween 20. For the immune reaction with anti-β-catenin Ab, cells were fixed with methanol for 10 min. IF detection of E-cadherin on cell lines was routinely performed on confluent cells to be sure to visualize stable cell-cell contacts. Indeed, in confluent monolayers, some SKOV3 cells also display E-cadherin expression. Samples were analyzed using an Eclipse TE2000-S microscope with a 40X 0.75NA PanFluor objective (Nikon, Tokyo, Japan). Images were acquired with ACT-1 software (Nikon). Confocal microscopy was carried out using a Leica TCS SP8 X confocal laser scanning microscope (Leica Microsystems GmbH, Mannheim, Germany). Images were acquired in the scan format 512 × 512 pixels in a single plane using a HC PL APO CS2 40X/1.30 oil-immersion objective and a pinhole always set to 1 Airy unit and analyzed using Leica LAS AF rel. 3.3 (Leica Microsystems GmbH) software. Images were processed using ImageJ and Adobe Photoshop softwares.

### Treatment with inhibitors

Cells were seeded into 96-well plates at a density of 3 × 10^3^cells/well. Twenty-four h after seeding, cells were exposed to increasing concentrations of roscovitine (Sigma-Aldrich) for up to 96 h. For drug combination studies, cisplatin alone or in combination with the CDK5 inhibitor, roscovitine (10 μM), was added 24 h after cell seeding. For 3D cytotoxicity, cisplatin and roscovitine, alone or in combination, were added to MCAs grown on Algimatrix™ 96-well plates for 5 days. In these experiments, mitochondrial activity was measured up to 96 h using CellTiter-Glo® Luminescent Cell Viability Assay. The combination index (CI) of drug treatment was established using the Chou and Talalay method [26] and CompuSyn software (Biosoft, Cambridge, UK). Cells were treated with 10 μM roscovitine or cisplatin, 3 or 1,5 μM respectively, alone or in combination for up to 48 h to evaluate cell viability with LIVE/DEAD™ Viability/Cytotoxicity Kit, for mammalian cells, performed according to the manufacturer’s instructions (ThermoFisher Scientific).

### Cell proliferation assay of 2D cultures

LZRS or PLEKHA7 cells were seeded at 47,500 cells/well in a 6-well plate for each experimental condition. At the end of each time point, cells were trypsinized, re-suspended in medium, immediately counted twice for each well, and the averages and standard deviations for four replicates were calculated for each condition. Cell cycle analysis was performed on EGF stimulated cells for 48 h of starved cells by flow cytometry upon propidium iodine staining as described [4].

### Soft agar assay

A 2 ml layer of 1% agar (wt/vol) in EMEM with 10% FBS was poured in 6-well plates. OAW42 cells transiently transfected with PLEKHA7 siRNA (Dharmacon, GE Healthcare) or with PLEKHA7-LZRSms-neo construct or transfected with the respective controls were suspended in 0.35% agar in EMEM with 10% FBS at a density of 10,000 cells/ml. Cell suspensions were poured on the top of the base layer and incubated at 37 °C in the presence of 5% CO_2_ for 15 days. The colonies were counted using an inverted microscope with a 10X 0,75 NA PanFluor objective (Nikon). Images were acquired with ACT-1 software (Nikon).

### In silico analysis of PLEKHA7 expression and survival analysis

Gene expression datasets publicly available on GEO repository (http://www.ncbi.nlm.nih.gov/geo) were considered. Nine datasets (GSE18520, GSE27651, GSE14001, GSE12172, GSE14407, GSE23391, GSE29450, GSE20565, GSE19352), profiled on the same array platform (Affymetrix U133 plus 2.0), were selected and reported data for ovarian surface epithelium (OSE), low malignant potential (LMP) tumors, low and highgrade tumors. They account for a total of 333 samples including: i) 45 OSE; ii) 38 LMP; iii) 57 low grade; 191 high grade tumors. Signal intensity was normalized within each individual dataset using Robust Multi-Array Average (RMA) tool. The datasets were integrated following a meta-analysis approach by applying analytical methods for data normalization and batch effect correction as described previously [27]. The log2 expression of PLEKHA7 identified by 242417_at Affymetrix probe set was retrieved. Kaplan-Meier Plotter (http://kmplot.com) was exploited for survival analyses.

### Statistical analysis

GraphPad Prism software (GraphPad Software, San Diego, CA) was used to analyze all data. Differences between mean values were determined by Student’s t and 2wayANOVA tests. Each experiment was performed at least three times for each condition; representative experiments are shown. Spearman correlation was applied to evaluate E-cadherin and EGFR protein correlation.

## Results

### E-cadherin membrane expression contributes to EOC MCA formation

**Fig. 1:**
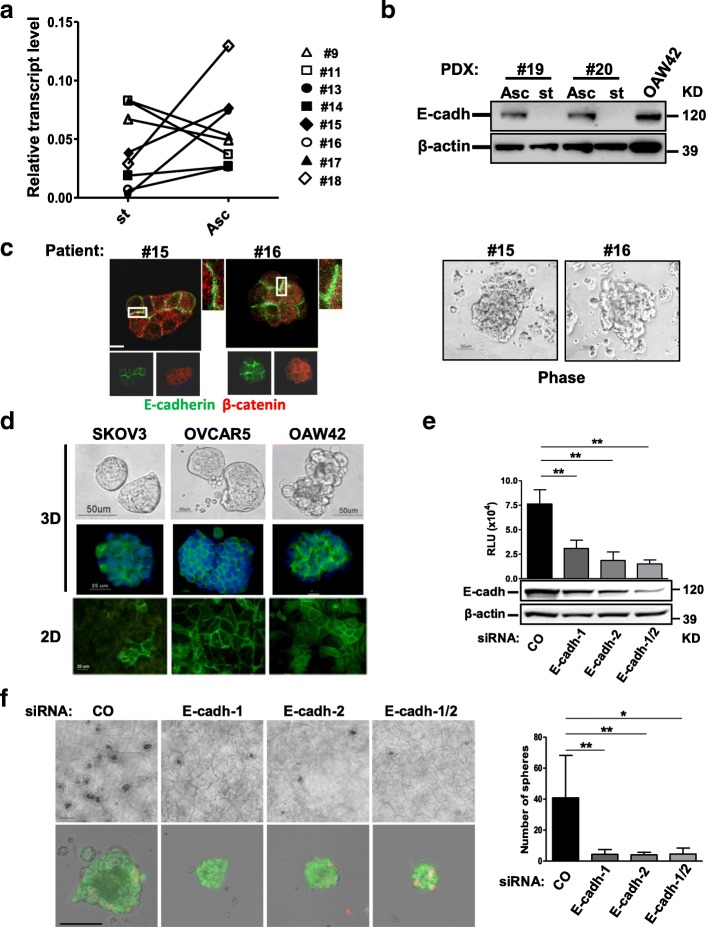
E-cadherin membrane expression contributes to EOC MCA formation. **a** Real-time RT-PCR showing the levels of E-cadherin transcript in freshly isolated matched solid peritoneal tumor masses (st) and ascites-derived MCAs (Asc) from eight HG-SOC patients. Results are presented as relative expression normalized to GAPDH mRNA levels. **b** Western blotting on total cell lysates from two pairs of MCAs (Asc) and solid tumors (st) of PDXs obtained from two different HG-SOC patients as reported in the Methods. β-actin was used as control of gel loading. **c** Representative images of MCAs from two HG-SOC patients. Left panel: staining with anti-E-cadherin and β-catenin Abs on MCAs from the same HG-SOC patients. Higher magnification images, corresponding to the highlighted white box are reported. Right panel: phase contrast microscopy performed on MCAs from two (#15 and #16) HG-SOC patients. **d** Upper panel: phase contrast and IF with anti-E-cadherin Ab (green) of 3D of SKOV3, OVCAR5, and OAW42 cells. Lower panel: IF with anti-E-cadherin Ab performed on confluent SKOV3, OVCAR5, and OAW42 cells (2D). **e** OAW42 cells transiently transfected with a control (CO) siRNA or with two E-cadherin siRNAs, separately (E-cadh-1, E-cadh-2) or pooled (E-cadh-1/2), and then grown as MCAs for 9 days. Upper panel: cell viability assay performed on silenced OAW42 MCAs. Lower panel: western blotting for evaluation of E-cadherin levels in OAW42 MCAs after 9 days of culture. A representative experiment is shown. Immunoblottings were performed with Abs against the proteins reported on the left. β-actin was used as a control for gel loading. **f** Left upper panel: representative phase contrast images of OAW42 MCAs obtained as above; bar, 100 μm. Left lower panel: phase contrast images of OAW42 MCAs obtained as above, dissociated from Algimatrix, and stain with LIVE/DEAD™ Viability/Cytotoxicity Kit. Merge of phase contrast, green (live cells) and red (dead cells) fluorescent images are shown. Single images are shown in Additional file 2: Figure S2a. Bar, 50 μm. Right panel: number of OAW42 MCAs (named spheres) obtained as above. Asterisks indicate statistically significant values by Student’s t test (*p* < 0.01)

We first assessed E-cadherin expression and localization in the two tumor cell populations that can co-exist in HG-SOC patients. We analyzed the expression of E-cadherin transcripts by real time RT-PCR on total RNA extracted from matched solid peritoneal tumor masses (st) and ascites-derived MCAs (Asc) from 8 HG-SOC patients. In 5 of 8 cases, the transcript level was higher in MCAs compared to the matched solid tumor mass (Fig. 1a). Although not statistically significant, this analysis indicated a trend towards higher E-cadherin expression in a subset of MCAs compared to the corresponding solid primary tumors. Western blotting of total cell lysates from solid peritoneal tumor masses and ascites-derived MCAs of two different HG-SOC PDXs confirmed higher levels of E-cadherin protein expression in MCAs in both cases (Fig. 1b). IF showed E-cadherin on the membrane at sites of cell-cell contact together with β-catenin in the MCAs (Fig. 1c, left panel). Immunohistochemical analysis, performed on sections from the corresponding solid EOC biopsies, showed E-cadherin staining on the cell membrane (Additional file 2: Figure S1), in agreement with previously published data [3–5].

To investigate the impact of membrane E-cadherin expression on EOC cells grown as MCAs, we set up an AlgiMatrix™ culture (named 3D in Fig. 1d) of EOC cell lines SKOV3, OVCAR5, and OAW42. All cell lines formed compact MCAs (upper panel), similar to those present in EOC ascites (see Fig. 1c, right panel). IF showed E-cadherin expression at the cell-cell contacts (Fig. 1d, middle panel). Interestingly, SKOV3 cells, which displayed membrane E-cadherin localization in only a few cells when grown as confluent monolayer on plastic (named 2D in Fig. 1d, lower panel), exhibited homogeneous membrane E-cadherin localization in 3D cultures. Next, we asked whether the presence of membrane E-cadherin was necessary for MCA formation. The capability of OAW42 cells to form MCAs in AlgiMatrix™ was evaluated after E-cadherin knockdown by transient transfection of two different siRNAs, used separately or pooled. Growth rate was significantly reduced in E-cadherin silenced cells compared to cells transiently transfected with a control siRNA (Fig. 1e, upper panel). E-cadherin expression, evaluated by western blotting on cell lysates prepared at the end of the experiment (day 9), resulted directly correlated (by Spearman, *r* = 0,88, *p* = 0,001) to cell numbers (Fig. 1e). MCAs from E-cadherin-silenced cells were indeed viable but smaller and significantly formed lower number of spheres (Fig. 1f). Only few cell debris were observed (Additional file 2: Figure S2a) likely due to the procedure necessary for Algimatrix dissolution. Accordingly, the size of E-cadherin silenced OAW42 MCAs grown in Matrigel® was also significantly smaller (Additional file 2: Figure S2b). In OVCAR5 cells E-cadherin silencing also negatively affected cell growth in a 2D system; no direct correlation with E-cadherin depletion was observed (Additional file 2: Figure S2c).

These data indicate that junctional E-cadherin contributes to EOC growth arguing that at least a subset of EOC MCAs present in ascites might rely on the increased expression of junctional E-cadherin for their growth.

### E-cadherin positively impinges EGFR activation

**Fig. 2:**
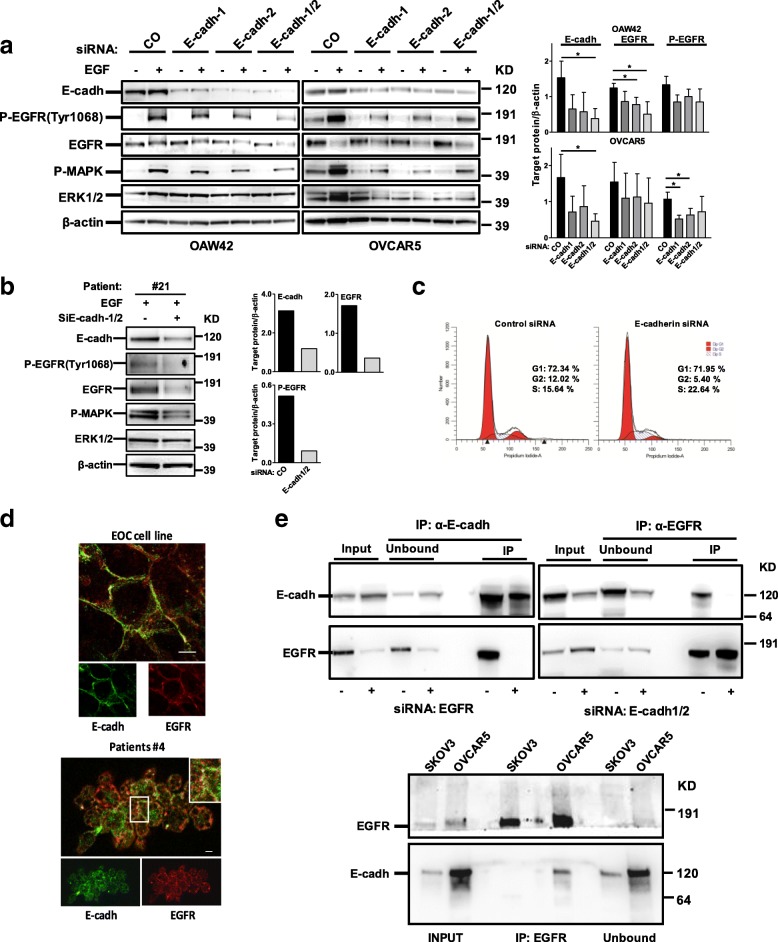
E-cadherin positively impinges EGFR activation. **a** Left panel: representative western blotting of three performed on lysates from OAW42 and OVCAR5 cells transiently transfected with a control (CO) siRNA or with two E-cadherin siRNAs, separately (E-cadh-1, E-cadh-2) or pooled (E-cadh-1/2). Cells were starved (−) for 24 h and then stimulated with EGF 20 ng/ml (+) for 15 or 30 min (OAW42 and OVCAR 5, respectively). β-actin was used as control for gel loading. Right panel: quantitative evaluation of E-cadherin, EGFR and P-EGFR on E-cadherin silenced cells. The graph reports the ratio between the target protein and β-actin from three different experiments performed on both OAW42 and OVCAR5 cells. **b** Left panel: western blotting on lysates from MCAs from HG-SOC patient #21 transiently transfected with a control (−) or a pool (+) of E-cadherin siRNAs (E-cadh-1/2). Cells were starved for 24 h and then stimulated with EGF 20 ng/ml overnight (+). Right panel: quantitative evaluation performed as Fig. 1a panel right. **c** Cell cycle analysis performed on OAW42 cells transiently transfected with a control (Control siRNA) or a pool of E-cadherin siRNAs (E-cadherin siRNA), starved 24 h and then stimulated with EGF 20 ng/ml for 48 h. Western blotting with anti-E-cadherin Ab to evaluate E-cadherin silencing on these experiments are reported in Additional file 2: Figure S2d. **d** Upper panel: confocal IF on fixed OAW42 cells performed with anti-E-cadherin (cadh, green) and -EGFR (red) Abs. Lower panel: Representative staining with anti-E-cadherin (cadh, green) and anti-EGFR (red) Abs on MCAs from HG-SOC patient #4. Enlarged detail in box is shown in the upper right side of the merge image. Bars, 10 μm. **e** Upper panel: IP performed with anti-EGFR or -E-cadherin (cadh) Abs on lysates from OAW42 cells. IPs were performed upon transient transfection of non silencing RNA or siRNA for E-cadherin or EGFR followed by IP with anti-EGFR or –E-cadherin, respectively, to test Abs specificity. Upon knockdown of the relevant protein, the complex E-cadherin/EGFR was not formed. Input, total cell lysates; Unbound, protein fraction not immunoprecipitated. Lower panel: IP performed with anti-EGFR Ab on lysates from 3D OVCAR5 and SKOV3 cells. Immunoprecipitated samples were analyzed by western blotting together with the unbound fraction. Immunoblottings were performed with Abs against the proteins reported on the left

To investigate whether E-cadherin could exert its role on EOC growth by supporting EGFR activation, transient E-cadherin silencing was performed on OAW42 and OVCAR5 cells, the latest considered an aggressive model of HG-SOC [28]. EGFR auto-phosphorylation at tyrosine (Tyr) 1068 upon EGF stimulation of control-siRNA cells increased in both cell lines (Fig. 2a, left panel) but it decreased upon E-cadherin silencing with both E-cadherin siRNAs, used either separately or pooled. E-cadherin silencing was also associated to a decrease of EGFR protein levels and Spearman correlation analysis revealed a significant positive correlation between E-cadherin and EGFR protein levels in both silenced cell lines [*r* = 0.6 (*p* = 0.04) and 0.8 (*p* = 0.02) in OAW42 and OVCAR5, respectively]. To be noted that in these cell lines EGF stimulation did not affect E-cadherin expression in control siRNA-treated and untreated cells (Additional file 2: Figure S2e), as reported previously [12]. The same results were also basically observed on the MCAs from patient #21 following 24 h stimulation with EGF to prevent cell suffering to excessive starvation. As well, ERK activation decreased (Additional file 1: Table S2). In EGF-stimulated control silenced cells EGFR was phosphorylated and upon E-cadherin knockdown both EGFR protein and phosphorylation levels decreased (Fig. 2b, Additional file 1: Table S2). Combined, these data highlight that E-cadherin depletion causes a decrease of net EGFR/ERK signaling. To investigate whether the observed decrease of the growth rate of E-cadherin silenced cells (Additional file 2: Figure S2d) was due to the decrease of EGF/EGFR activation (Additional file 2: Figure S2d), a cell cycle analysis was performed on starved cells upon stimulation with EGF for 48 h. S phase block and a consequent reduction of the G2/M phase were observed in EGF stimulated E-cadherin silenced cells respect to control-silenced cells with no apparent sub-G0 peak (Fig. 2c) consistent with a diminished EGF-dependent growth potential upon E-cadherin depletion. In addition, in OAW42 cells, while E-cadherin silencing was associated to a slight N-cadherin increase, EGFR activation was not associated to a decrease of E-cadherin (Additional file 2: Figure S2d and e), indicating that EGF stimulation is not associated to cadherin switch as was previously observed in other cell lines [12, 13].

Confocal IF performed on OAW42 cells with anti-E-cadherin and anti-EGFR Abs showed membrane staining for both E-cadherin and EGFR (Fig. 2d, upper panel) with 43% co-localization. The same pattern of E-cadherin and EGFR staining was also observed on the membrane of MCAs of HG-SOC patients (a representative image is shown in Fig. 2d, lower panel).

IP experiments performed with anti-EGFR or E-cadherin Abs on OAW42 cell lysates demonstrated that EGFR and E-cadherin form a biochemical complex in these tumor cells (Fig. 2e, upper panel). To test the specificity of the relevant Abs, IPs were performed upon silencing of E-cadherin or EGFR and immunoprecipitation with anti-EGFR and –E-cadherin, respectively, clearly demonstrating that upon the knockdown of the relevant protein, E-cadherin/EGFR complex was not detected in the immunoblotting. Accordingly, in AlgiMatrix™ OVCAR5 MCAs, but not in E-cadherin-low expressing SKOV3 cells, a complex between EGFR and E-cadherin was observed following IP with anti-EGFR Ab (lower panel).

Combined, these data demonstrate that E-cadherin can associate with EGFR in HG-SOCs, affecting overall receptor activation.

### EGFR/CDK5 signaling is activated in E-cadherin-expressing cells and can be inhibited by roscovitine

**Fig. 3:**
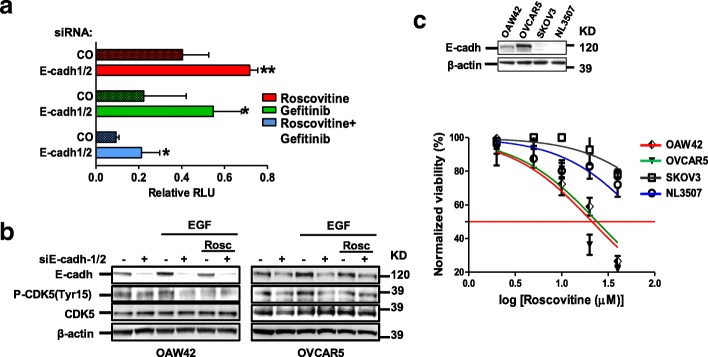
EGFR/CDK5 signaling is activated in E-cadherin-expressing cells and can be inhibited by roscovitine. **a** Proliferation assay performed on control (CO) or E-cadherin silenced OAW42 cells (E-cadh-1/2) grown in the presence of EGF and treated with roscovitine alone (20 μM), or gefitinib (10 μM) or with both drugs. Asterisks indicate statistically significant values by Student’s t test. A representative experiment is shown of three performed. **b** Western blotting on total cell lysates from OAW42 and OVCAR5 cells transiently transfected with control (−) or a pool (+) of E-cadherin siRNAs (siE-cadh-1/2), starved and then stimulated with EGF 20 ng/ml and treated with roscovitine. Immunoblottings were performed with Abs against the proteins reported on the left. β-actin was used as control of gel loading. **c** Upper panel: western blotting on total cell lysates from OVCAR5, OAW42, SKOV3 and NL3507 cells. Immunoblottings were performed with Abs against the proteins reported on the left. β-actin was used as control of gel loading. Lower panel: cell viability assay on OVCAR5, OAW42, SKOV3 and NL3507 cells treated with roscovitine (2, 5, 10, 20, 40 μM) up to 96 h. Each point represents the mean of three replicates. Error bars, SD

To analyzed E-cadherin/EGFR dependency of CDK5 activation, the efficacy of CDK5 inhibitor roscovitine to inhibit EOC cell growth was tested alone or together with EGFR inhibitor gefitinib in E-cadherin silenced OAW42 cells (Additional file 2: Figure S2c) grown in presence of EGF. Roscovitine or gefitinib alone or their combination was significantly less effective in E-cadherin silenced cells (Fig. 3a). Indeed, upon E-cadherin knockdown in OAW42 and OVCAR5 cells treated with EGF, CDK5 phosphorylation on Tyr 15 (P-CDK5) was reduced by E-cadherin silencing on both cell lines (Fig. 3b). Treatment with roscovitine decreased P-CDK5 of control siRNA-treated cells to the same extent as silencing of E-cadherin and provided no further inhibition of P-CDK5 in E-cadherin knockdown cells (Fig. 3b) indicating that the CDK5 inhibitor is effective in cells expressing E-cadherin. Accordingly, roscovitine effect upon treatment with increasing concentrations (from 2 to 40 μM) was monitored on four EOC cell lines, expressing or not expressing E-cadherin (Fig. 3c, upper panel). OAW42 and OVCAR5 cells, which express higher levels of E-cadherin, were sensitive to roscovitine inhibition with an IC_50_ of 24 and 21 μM, respectively (lower panel). In contrast, SKOV3 and NL3507 cells, expressing almost undetectable levels of E-cadherin, were only marginally sensitive to roscovitine treatment (IC_50_ was not reached). Altogether, these results indicate that E-cadherin plays a role in EGFR/CDK5 activation and the CDK5 inhibitor roscovitine may be selectively effective in E-cadherin-expressing EOC cells.

### Cisplatin susceptibility in E-cadherin-expressing EOC cells is enhanced by combination with CDK5 inhibitor

**Fig. 4:**
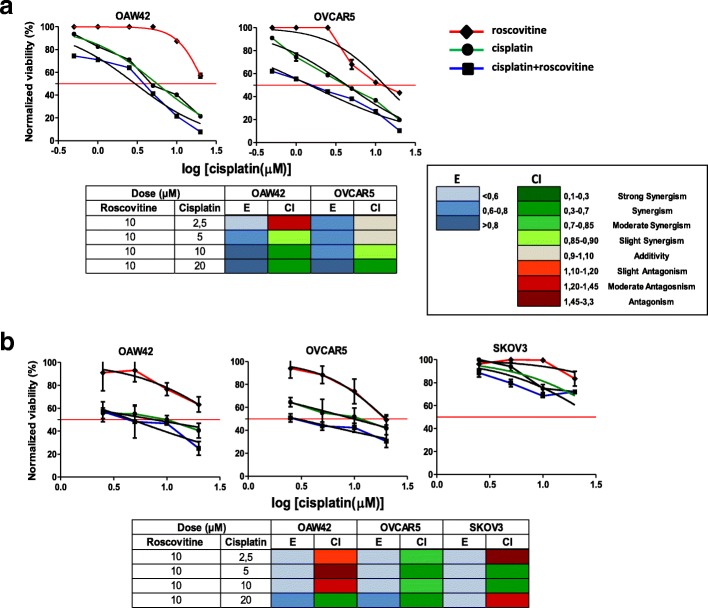
Cisplatin susceptibility in E-cadherin-expressing EOC cells is enhanced by combination with CDK5 inhibitor. **a** Dose-response curves of OAW42 and OVCAR5 cells treated with increasing concentration of roscovitine or cisplatin or alone (0.625, 1.25, 2.5, 5, 10, 20 μM) or in combination with 10 μM (the dose below the IC_50_ in these cells) roscovitine up to 96 h. The red line shows the dose-response curve to roscovitine alone. **b** Dose-response curves of OAW42, OVCAR5 and SKOV3 cells grown as MCA in Algimatrix™ treated treated as above. Cell viability was measured by CellTiter-Glo® Luminescent Cell viability assay. Tables below each panel: drug interaction analysis evaluated by the Chou and Talalay method. Effect and combination index are reported in the lower right panel A; color-coded fraction represents the effect values (E) and the combination index (CI) at the indicated drug combination doses

To investigate whether roscovitine could exert a synergistic inhibitory effect with platinum compounds, which are the chemotherapeutics of choice for EOC patients [29], E-cadherin-expressing OAW42 and OVCAR5 cells were treated with increasing concentrations of cisplatin alone or in combination with a fixed concentration of roscovitine (10 μM). In both OAW42 and OVCAR5 cells, cisplatin was able to inhibit the proliferation rate; in the presence of roscovitine, the IC_50_ for cisplatin decreased from 7 to 3 μM and from 4 to 1 μM, respectively (Fig. 4a). Drug combination performed using the Chou and Talalay method unveiled synergism at high cisplatin doses for both cell lines (Fig. 4a, lower panel). Cell viable assay clearly showed cell death of both cell lines treated with cisplatin plus roscovitine (Additional file 2: Figure S3).

We also tested the efficacy of roscovitine on 3D AlgiMatrix™ cultures of OAW42, OVCAR5, and SKOV3 cells. As described for adherent cells, E-cadherin-expressing OAW42 and OVCAR5 MCAs were more sensitive to the combination of cisplatin and roscovitine (Fig. 4b, upper panel), and the effect was still synergistic (lower panel). In SKOV3 MCAs that express low levels of E-cadherin, the effect value was below 0.6 and the IC_50_ was not reached even with combined treatment.

The major implication of these observations is that in E-cadherin-expressing EOC cells combined treatment with a drug able to inhibit EGFR/CDK5 activation increases sensitivity to cisplatin.

### Impact of PLEKHA7 on E-cadherin-mediated EGFR signaling activation

**Fig. 5:**
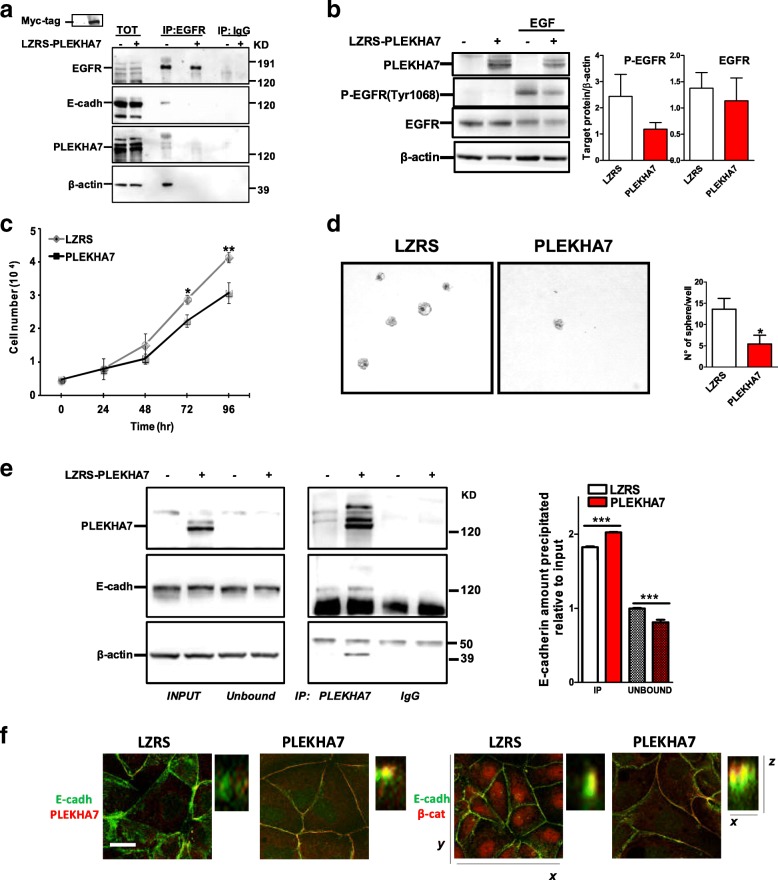
Impact of PLEKHA7 on E-cadherin-mediated EGFR signaling activation. **a** IP performed with anti-EGFR Ab on lysates from OAW42 cells transiently transfected with an empty LZRS (−) or with a LZRS- PLEKHA7 vector (+). Normal rabbit (IgG) serum was used as negative control. Immunoprecipitated samples were analyzed by western blotting with Abs against the proteins reported on the left. The inset above indicates the Myc-tag overexpression corresponding to PLEKHA7 expression. **b** Left panel: western blotting on total cell lysates from starved empty LZRS vector (−) or LZRS-PLEKHA7 infected OAW42 cells (+) starved and then stimulated with EGF 20 ng/ml for 30 min. Right panel: graph reporting the amount of P-EGFR anf EGFR in EGF stimulated cells evaluated in three different immunoblottings. **c** Proliferation assay of empty LZRS vector or PLEKHA7 infected OAW42 cells. Asterisks indicate statistically significant values evaluated with one-way Anova. **d** Left: representative phase contrast images of infected OAW42 cells grown in soft agar for 10 days. Three replicates for each condition were done. Right panel: graph reporting the number of clones/well. Asterisk indicates significant values (*p*˂0.05). **e** Left panel: IP with anti-PLEKHA7 Ab on lysates from LZRS vector (−) or LZRS-PLEKHA7 (+) OAW42 cells. Immunoblottings were performed with Abs against the proteins reported on the left. Right panel: quantitative analysis of E-cadherin immunoprecipitated with PLEKHA7 Ab or present in the unbound fraction evaluated in three different experiments as the ratio between the immunoprecipitated (IP) or not immunoprecipitated (Unbound) and the total input. Asterisks indicate significant values (*p*˂0.001). **f** Confocal IF performed on infected OAW42 cells immunostained with E-cadherin (E-cadh) together with PLEKHA7, or -β-catenin (cat) Abs. The panel reports the 0.5 μm stacks acquired from the bottom to the top of the cells. Merge images are shown. Bar, 20 μm for xy images; bar, 3 μm for xz images

It has been previously shown that the growth suppressor role of E-cadherin in polarized epithelia depends on the expression of PLEKHA7 [9]. Thus, we investigated whether PLEKHA7 could also affect E-cadherin/EGFR crosstalk in EOC cells. Among EOC cell lines, only OAW42 and OVCAR5 cells showed a detectable expression of the 145 kDa PLEKHA7 isoform (Additional file 2: Figure S4a) with a localization on cell membrane (Additional file 2: Figure S4b). Based on the observation that PLEKHA7 is low in EOC cells, we investigated whether PLEKHA7 overexpression could modulate E-cadherin behavior in EOC cells. In OAW42 cells PLEKHA7 was transiently transfected and an IP was performed using anti-EGFR Ab. PLEKHA7 overexpression inhibited EGFR-E-cadherin complex formation (Fig. 5a), whereas in control LZRS-transfected OAW42 cells EGFR formed a complex with E-cadherin and actin.

Stably PLEKHA7 infected OAW42 cells were then used to test the hypothesis that the dissociation of E-cadherin from EGFR following PLEKHA7 overexpression affects E-cadherin-mediated EGFR activation. P-EGFR levels decreased in lysates of these cells upon EGF stimulation (Fig. 5b). Conversely, PLEKHA7 highly expressing Caco2 cells stably infected with a shPLEKHA7 vector [9] showed EGFR phosphorylation soon after 5 min EGF stimulation and after 15 min reached up to 20 times higher EGFR phosphorylation than mock infected cells (Additional file 2: Figure S4c) thus further demonstrating the biological relationship among E-cadherin, PLEKHA7 and EGFR.

Indeed, PLEKHA7 infected OAW42 cells showed a significant decrease in growth potential in 2D cultures (Fig. 5c). Similarly, in 3D AlgiMatrix™ cultures, smaller and significantly fewer MCAs were observed upon PLEKHA7 overexpression compared to control cells (Additional file 2: Figure S5b). PLEKHA7-overexpressing cells displayed a 60% reduction in their capacity to form colonies in soft agar (Fig. 5d), indicating a less tumorigenic phenotype.

To investigate whether in tumor cells PLEKHA7 can redirect E-cadherin to apical cell-cell junctions as observed for normal epithelial cells [30], an IP for PLEKHA7 was performed. Anti-PLEKHA7 immunoprecipitated lysates from PLEKHA7 overexpressing cells contained higher levels of E-cadherin, as well as higher levels of actin, compared to lysates from LZRS vector control cells suggesting a stronger association of the E-cadherin complex with the submembrane cortical cytoskeleton (Fig. 5e). Accordingly, E-cadherin not complexed to PLEKHA7 (Unbound) was significantly less in PLEKHA7 overexpressing cells (right panel). Confocal IF showed that in OAW42 cells overexpressing PLEKHA7 E-cadherin and PLEKHA7 co-localized and were strongly expressed at apical sites of cell-cell contact (Fig. 5f, see Additional file 2: Figure S5a for single immunoreactivity). Confocal IF of E-cadherin together with anti β-catenin further confirmed focal co-localization at the apical sites in cells overexpressing PLEKHA7. Furthermore, β-catenin is exclusively at the cell membrane in PLEKHA7 overexpressing OAW42 cells, which is suggestive of a less aggressive phenotype compared to LZRS vector control cells that also exhibit nuclear β-catenin localization [31].

These observations demonstrate that PLEKHA7 and E-cadherin co-localize at the apical cell sites in PLEKHA7 overexpressing cells, likely recapitulating the ZAs of normal epithelial cells. Accordingly, the presence of higher levels of PLEKHA7 inhibits E-cadherin/EGFR association thus negatively affecting growth and tumorigenic potential of EOC cells.

### Clinical impact of PLEKHA7 in EOC patients

**Fig. 6:**
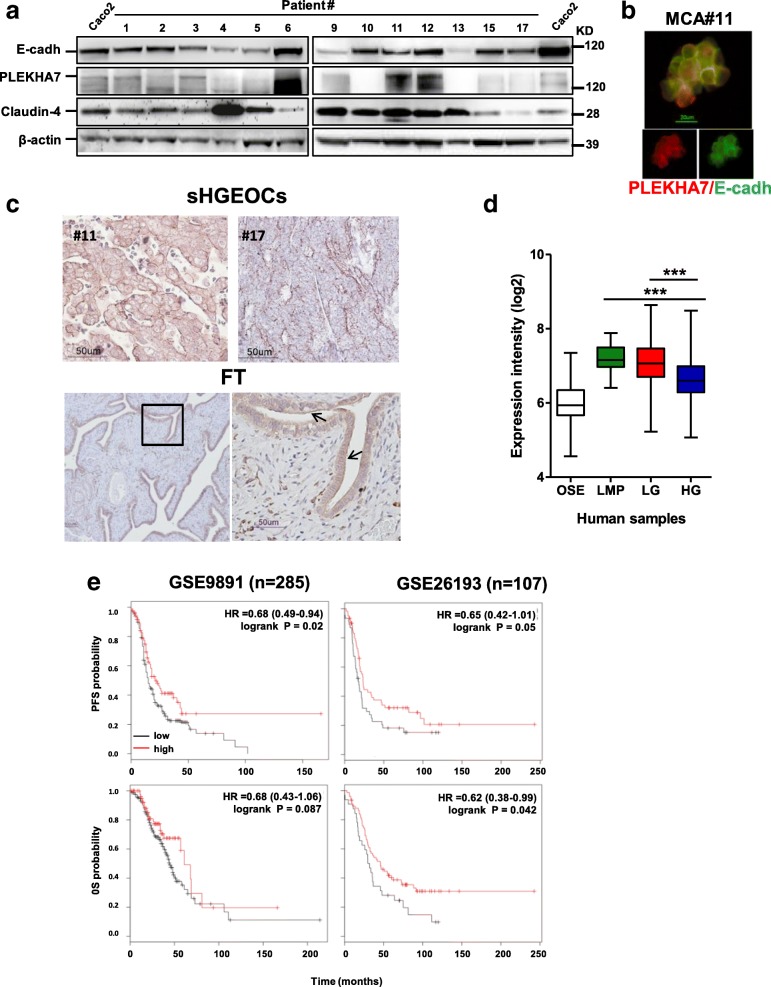
Clinical impact of PLEKHA7 in EOC patients*.* **a** Western blotting on total cell lysates from MCAs present in ascites from HG-SOC patients (*n* = 13). Caco2 lysates were used as positive control of PLEKHA7 expression. Claudin-4 was included as epithelial marker. Immunoblottings were performed with Abs against the proteins reported on the left. β-actin was used as control of gel loading. **b** Representative IF performed on a fixed sample of HG-SOC MCAs (sample #11) with anti-E-cadherin (cadh) and anti-PLEKHA7 Abs. **c** Representative images of the IHC performed with anti-PLEKHA7 Ab on HG-SOCs. The reaction on the fallopian tube epithelium (FT) was considered as a positive control. The empty black box in the left panel highlights the image reported in the right panel at higher magnification. **d** Meta-analysis, as described in Methods section, for evaluation of PLEKHA7 expression intensity in OSE and EOCs of different histotypes. Asterisks indicate statistically significant values by Student’s t test (*p* ˂ 0.001). **e** Kaplan-Meyer curves reporting the PFS and OS analyses on patient selected for PLEKHA7 expression

To evaluate the impact of PLEKHA7 in EOC patients, we analyzed the expression of PLEKHA7 and E-cadherin in MCAs from HG-SOC patients. Among 13 lysates, the full length 145 kDa isoform of PLEKHA7 was clearly expressed in 5 samples (patient #1, #3, #6, #11 and #12 of Fig. 6a) in association with E-cadherin. All samples showed E-cadherin expression and, when co-expressed, PLEKHA7 and E-cadherin were detected at the site of cell-cell contacts (representative image of MCAs from patient #11 in Fig. 6b). Immunohistochemical analysis performed on the same HG-SOC cases of Additional file 2: Figure S1 showed low PLEKHA7 expression in all samples and virtually no membrane localization (Fig. 6c, upper panel), contrary to the apical expression observed in normal tubal epithelium (lower panel).

To investigate the hypothesis that PLEKHA7 characterizes more differentiated and less aggressive EOC, we first analyzed PLEKHA7 expression in tumors with different histology and aggressiveness. A meta-analysis of PLEKHA7 expression intensity was performed on datasets of gene expression reporting data of 333 samples (Additional file 1: Table S3). A significant lower PLEKHA7 expression intensity was observed in HG-SOC respect to both low malignant potential (LMP) and low grade (LG) EOCs (Fig. 6d). Being not frankly epithelial [32], OSE show the lowest PLEKHA7 expression.

The correlation of PLEKHA7 expression and progression-free and overall survivals were analysed in two public available datasets [33, 34] containing gene expression data from 285 and 107 EOC patients, respectively. In both datasets, higher PLEKHA7 gene expression was significantly associated to longer progression free survival (PFS) (log-rank test, *p* = 0.02 and 0.05, respectively) (Fig. 6e, upper panels); longer overall survival (OS) significantly associated to higher PLEKHA7 gene expression in the dataset GSE26193 (lower panel).

The latest data indicate that PLEKHA7 is lost or delocalized in EOCs and its expression might be prognostically relevant.

## Discussion

This study unravels a mechanism through which E-cadherin promotes a pro-tumorigenic effect in EOCs. We provide several evidences of the association between E-cadherin and EGFR and showed for the first time that low or absent PLEKHA7 expression in EOCs allows E-cadherin to form complexes with EGFR at the cell surface. Upon PLEKHA7 overexpression, the association of E-cadherin to EGFR is lost and subsequent EGFR activation inhibited. The relevance of these findings to human disease is further highlighted by evidence of low levels of PLEKHA7 in HG-SOC patients’ tumors. Patients retaining higher PLEKHA7 transcript levels are characterize by better outcomes.

Herein, we strengthen previous findings [9] on the growth-suppressive role of PLEKHA7 even in malignant non-polarized cells and demonstrated that restoring PLEKHA7 expression results in reduced E-cadherin-EGFR signaling. PLEKHA7, a component of the ZA in polarized epithelia, is either mis-localized or lost in breast and renal carcinomas, and often not associated to E-cadherin loss [9, 35]. In non-transformed polarized epithelia, the association between PLEKHA7 and p120 catenin has been shown to determine a growth-suppressive role for E-cadherin associated with cell-cell contacts at the apical ZA, whereas a cell growth promoting role is exerted by the E-cadherin-p120 catenin complex upon PLEKHA7 loss [9]. Our in vitro data demonstrate in EOCs that E-cadherin forms a complex with EGFR, thus contributing to growth-promoting signaling. E-cadherin/EGFR crosstalk might be peculiar of transformed cells since tubal epithelial cells, from which HG-SOC originate [36], express E-cadherin but not EGFR (https://www.proteinatlas.org/ENSG00000146648-EGFR/tissue and data not shown), and ovarian surface epithelial cell, the other cells of EOC origin [37], only express N-cadherin. Interestingly, the approach using E-cadherin knockdown indicates a significant correlation between E-cadherin and EGFR protein levels and we show similar relationship in an EOC patient-derived sample. These data support the idea that E-cadherin alone or together with the junctional partners might contribute to membrane EGFR stability. All these aspects await further investigation.

The peculiar role of E-cadherin has been also described for other tumors. Recently, two reports have presented evidence that EMT is not a prerequisite for metastasis formation in mouse models of breast and pancreatic cancer [38, 39]. In these studies, although some cells of the primary tumor have undergone EMT, the metastases-containing cells are still expressing E-cadherin. Given the ability of PLEKHA7 to modulate pro- vs. anti-tumorigenic E-cadherin function, it would be interesting to evaluate whether PLEKHA7 can regulate E-cadherin behavior in these cancer types. Hence, the biological impact of E-cadherin/EGFR complex at the junctions in relation with PLEKHA7 await further investigations in tumor cells others than EOCs.

E-cadherin requirement for MCA formation is supported by the observation that EOC cell lines growing in 3D increase E-cadherin expression, including SKOV3 cells that display slightly E-cadherin expression only when highly confluent as monolayer. The leading role of E-cadherin in sustaining growth, maintenance, and resistance to chemotherapy in 3D models of EOC cells was recently reported by Xu et al. who suggested E-cadherin as therapeutic target [40] and Latifi et al. had reported a list of genes, which include E-cadherin, that characterizes chemo-resistant EOC MCAs [41]. In this regard, experiments on EOC cells with peptidomimetics [42, 43] and small chemical inhibitors to E-cadherin are ongoing in our laboratory.

Recent data showed that N-cadherin-expressing, EOC MCAs, but not those expressing E-cadherin, are responsible for intraperitoneal cell seeding upon mesothelial cell clearance [44, 45]. Although we show here that the majority of EOC MCAs tested express E-cadherin, it is likely that during peritoneal dissemination EOC cells of MCAs undergo to EMT-like due to their plasticity [45, 46]. Furthermore, other groups, together with ours, have already documented the expression of E-cadherin in advanced-stage HG-SOC [3, 4, 47, 48].

In malignant cells, E-cadherin allows cell-to-cell communication and, in addition, can act as a linker for membrane receptors and cytoplasmic signaling molecules such as PI3K, as we demonstrated previously [4], or EGFR, as shown in the present work. Although EGFR is expressed in the majority of EOCs [49], and about 37% of EOCs present EGFR gene amplification associated to worst prognosis [10], EGFR inhibitors resulted not efficacious in curing EOC patients [16] suggesting that a complete landscape of the EGFR-activated signaling remains to be better clarified. We here demonstrate that EOC MCAs take advantage of associated E-cadherin-dependent EGFR/CDK5 signaling for growing in the malignant ascites and ex vivo analysis performed on human HG-SOC samples indicate that E-cadherin-mediated EGFR/CDK5 signaling might be activated in a subset of MCAs. In line with this, the use of 3D cultures, mimicking EOC MCA growth, allowed us to assess the synergistic effects of the CDK5 inhibitor roscovitine on the efficacy of cisplatin, the standard of care for these patients suggesting the combined use of cisplatin and CDK5 inhibitor roscovitine as new therapeutic approach for EOC patients. Future efforts will be devoted to assess the efficacy of this therapeutic approach in the most appropriate preclinical in vivo models and to also identify those EOC patients who would likely benefit of drugs inhibiting E-cadherin-dependent EGFR/CDK5 activation.

## Conclusion

We define in EOCs E-cadherin as a tumor enhancer that positively contributes to EGFR-promoting growth signaling due to loss of PLEKHA7 expression. This mechanism appears to be especially relevant for EOC patients in whom the peritoneal spreading of malignant cells requires that tumor cells, detached from the solid masses, can grow and/or survive in ascites as MCAs. Clinically, PLEKHA7 emerges as a possible marker for less aggressive EOC tumors. Our analysis also suggests that EGFR/CDK5 signaling pathway activated in E-cadherin-expressing EOC cells represent novel targets for therapies, at least in a subset of EOC patients not responding to EGFR inhibitors.

## Additional files

Additional file 1: **Table S1.** List of antibodies used in this study. **Table S2.** Quantitative evaluation of P-MAPK on E-cadherin silenced cells stimulated with EGF 20 ng/ml. The table reports the ratio between the target protein and β-actin, as the percentage of the control, from three different experiments performed on both OAW42 and OVCAR5 cells of Fig. 2a and from Fig. 2b. **Table S3.** Selected EOC samples from the publicly available datasets analyzed in the present study. (PDF 83 kb)

Additional file 2: **Figure S1.** IHC with anti-E-cadherin on: upper panel, FFPE sections from fallopian tubal epithelium; and lower panel, eight FFPE samples of solid masses from HG-SOC patients. Control, a section only processed with the secondary antibody. Bar, 50 μm. **Figure S2a.** Representative phase contrast images of OAW42 MCAs and evaluation of live/dead cells; bar, 50 μm. The empty box highlights the image reported in Fig. 1f. **b**. Upper panel: representative phase contrast images of MCAs of control (CO) and E-cadh siRNA-treated OAW42 cells grown in Matrigel® for 6 days. Lower panel: measurement of OAW42 MCA area using ImageJ software. **c**. Control (CO) or E-cadherin siRNA-treated OVCAR5 cells. Upper panel: cell viability assay performed on silenced OVCAR5 cells; the number of cells was evaluated. Lower panel: E-cadherin levels in OVCAR5 cells after 5 days of culture. **d**. E-cadherin levels in treated cells of Fig. 2c. Control, (CO) or pooled E-cadherin siRNA. **e**. Western blotting on lysates from OAW42 starved (−) or EGF treated cells. **Figure S3.** Representative phase contrast images or fluorescent marked OAW42 and OVCAR5 live/dead cells; bar, 100 μm. **Figure S4a.** Western blotting on total cell lysates from six EOC cell lines. **b**. IF on fixed Caco2, OAW42, and OVCAR5 cells. **c**. Upper panel: representative western blotting on lysates from Caco2 cells infected with a control (NT) or with PLEKHA7 shRNA (shPLEKHA7). Starved cells (−). Lower left panel: western blotting with anti-PLEKHA7 Ab. Lower right panel: quantitative P-EGFR/EGFR ratio on PLEKHA7 silenced cells as above. **Figure S5a.** Confocal IF performed on LZRS or LZRS-PLEKHA7 infected OAW42 cells. Bar, 20 μm. The panel reports the stacks with single Ab of the merge images of Fig. 5d. **b**. Left panel: representative phase contrast images of LZRS or PLEKHA7 OAW42 MCAs grown in Algimatrix™. Right panel: cell viability assay of cells extracted from the sponge. (PDF 791 kb)
