# Supplementary material for: Simultaneous E-cadherin and PLEKHA7 expression negatively affects E-cadherin/EGFR mediated ovarian cancer cell growth
Source: J Exp Clin Cancer Res. 2018 Jul 11;37:146. doi: 10.1186/s13046-018-0796-1 (PMC6042237; doi:10.1186/s13046-018-0796-1)
Supplement: Supplementary file 1 — Table S1. List of antibodies used in this study. Table S2. Quantitative evaluation of P-MAPK on E-cadherin silenced cells stimulated with EGF 20 ng/ml. The table reports the ratio between the target protein and β-actin, as the percentage of the control, from three different experiments performed on both OAW42 and OVCAR5 cells of Fig. 2a and from Fig. 2b. Table S3. Selected EOC samples from the publicly available datasets analyzed in the present study. (PDF 83 kb) [file 13046_2018_796_MOESM1_ESM.pdf]

Supplementary Table 1. List of antibodies used in this study.

Primary antibodies	Source	Catalog #	Dilution for IF	Dilution for WB	Dilution for IHC
EGFR (rabbit)	Cell Signaling Technology	2232		1:1000	
EGFR (MINT-5) (mouse)	Tosi et al. 1995		1:50		
P-EGFR(Tyr1068) (mouse)	Cell Signaling Technology	2236S		1:1000	
P-CDK5(Tyr15) (rabbit)	Abcam	TA312175		1:500	
CDK5 (mouse)	Santa Cruz Biotechnology	sc-6247		1:500	
β-actin (rabbit)	Sigma-Aldrich	A 2066		1:400	
E-cadh (rabbit)	Santa Cruz Biotechnology	sc-7870		1:200	1:200
E-cadh (mouse)	Invitrogen	13-1700	1:500		
claudin-4 (mouse)	Invitrogen	329400		1:500	
PLEKHA7 (rabbit)	Sigma-Aldrich	HPA038610	1:200	1:500	1:250
β-catenin	Sigma-Aldrich	C2206	1:300		

Supplementary Table 2. Quantitative evaluation of P-MAPK on E-cadherin silenced cells stimulated with EGF 20 ng/ml. The table reports the ratio between the target protein and β-actin, as the percentage of the control, from three different experiments performed on both OAW42 and OVCAR5 cells of Fig. 2a and from Fig. 2b.

SiRNA:	Cell line/ Patient sample				
	OAW42		OVCAR5		Patient #21
	Average	Standard deviation	Average	Standard deviation	
Co	100,00	1,42E-14	100,00	1,00E-14	100,00
E-cadh-1	67,35	4,07E+00	79,20	3,18E+01	
E-cadh-2	67,68	1,75E+01	46,20	2,35E+01	
E-cadh-1/2	70,70	2,78E+01	52,30	2,29E+01	54,59

Supplementary Table 3. Selected EOC samples from the publicly available datasets analyzed in the present study.

DATASETS	Reference	OSE n=47	EOC		
			**LMP** n=38	**LG** n=57	**HG** n=191
GSE18520	Mok SC, Bonome T, Vathipadiekal V, Bell A et al. *Cancer Cell* 2009 Dec 8;16(6):521-32	10	-	-	53
GSE27651	King ER, Tung CS, Tsang YT, Zu Z et al. *Am J Surg Pathol* 2011 Jun;35(6):904-12.	6	8	13	22
GSE14001	Tung CS, Mok SC, Tsang YT, Zu Z et al. Mod Pathol 2009 Sep;22(9):1243-50.	3	-	10	10
GSE12172	Anglesio MS, Arnold JM, George J, Tinker AV et al. *Mol Cancer Res* 2008 Nov;6(11):1678-90.	-	30	13	45
GSE14407	Bowen NJ, Walker LD, Matyunina LV, Logani S et al. BMC Med Genomics 2009 Dec 29;2:71.	12	-	-	-
GSE23391	Shahab SW, Matyunina LV, Mezencev R, Walker LD et al. PLoS One 2011;6(7):e22508.	5	-	-	-
GSE29450	Stany MP, Vathipadiekal V, Ozbun L, Stone RL et al. *PLoS One*2011;6(7):e21121.	10	-	-	-
GSE20565	Meyniel JP, Cottu PH, Decraene C, Stern MH et al. BMC Cancer 2010 May 21;10:222.	-	-	18	47
GSE19352	Iorio E, Ricci A, Bagnoli M, Pisanu ME et al. Cancer Res 2010 Mar 1;70(5):2126-35.	1	-	3	14
GSE9891	Tothill RW, Tinker AV, George J, Brown R et al. Clin Cancer Res 2008 Aug 15;14(16):5198-208.	-	18	11	256
GSE26193	Mateescu B, Batista L, Cardon M, Gruosso T et al *Nat Med*2011 Nov 20;17(12):1627-35.	-	-	7	100
