# Supplementary material for: Simultaneous E-cadherin and PLEKHA7 expression negatively affects E-cadherin/EGFR mediated ovarian cancer cell growth
Source: J Exp Clin Cancer Res. 2018 Jul 11;37:146. doi: 10.1186/s13046-018-0796-1 (PMC6042237; doi:10.1186/s13046-018-0796-1)
Supplement: Supplementary file 2 — Figure S1. IHC with anti-E-cadherin on: upper panel, FFPE sections from fallopian tubal epithelium; and lower panel, eight FFPE samples of solid masses from HG-SOC patients. Control, a section only processed with the secondary antibody. Bar, 50 μm. Figure S2a. Representative phase contrast images of OAW42 MCAs and evaluation of live/dead cells; bar, 50 μm. The empty box highlights the image reported in Fig. 1f. b. Upper panel: representative phase contrast images of MCAs of control (CO) and E-cadh siRNA-treated OAW42 cells grown in Matrigel® for 6 days. Lower panel: measurement of OAW42 MCA area using ImageJ software. c. Control (CO) or E-cadherin siRNA-treated OVCAR5 cells. Upper panel: cell viability assay performed on silenced OVCAR5 cells; the number of cells was evaluated. Lower panel: E-cadherin levels in OVCAR5 cells after 5 days of culture. d. E-cadherin levels in treated cells of Fig. 2c. Control, (CO) or pooled E-cadherin siRNA. e. Western blotting on lysates from OAW42 starved (−) or EGF treated cells. Figure S3. Representative phase contrast images or fluorescent marked OAW42 and OVCAR5 live/dead cells; bar, 100 μm. Figure S4a. Western blotting on total cell lysates from six EOC cell lines. b. IF on fixed Caco2, OAW42, and OVCAR5 cells. c. Upper panel: representative western blotting on lysates from Caco2 cells infected with a control (NT) or with PLEKHA7 shRNA (shPLEKHA7). Starved cells (−). Lower left panel: western blotting with anti-PLEKHA7 Ab. Lower right panel: quantitative P-EGFR/EGFR ratio on PLEKHA7 silenced cells as above. Figure S5a. Confocal IF performed on LZRS or LZRS-PLEKHA7 infected OAW42 cells. Bar, 20 μm. The panel reports the stacks with single Ab of the merge images of Fig. 5d. b. Left panel: representative phase contrast images of LZRS or PLEKHA7 OAW42 MCAs grown in Algimatrix™. Right panel: cell viability assay of cells extracted from the sponge. (PDF 791 kb) [file 13046_2018_796_MOESM2_ESM.pdf]

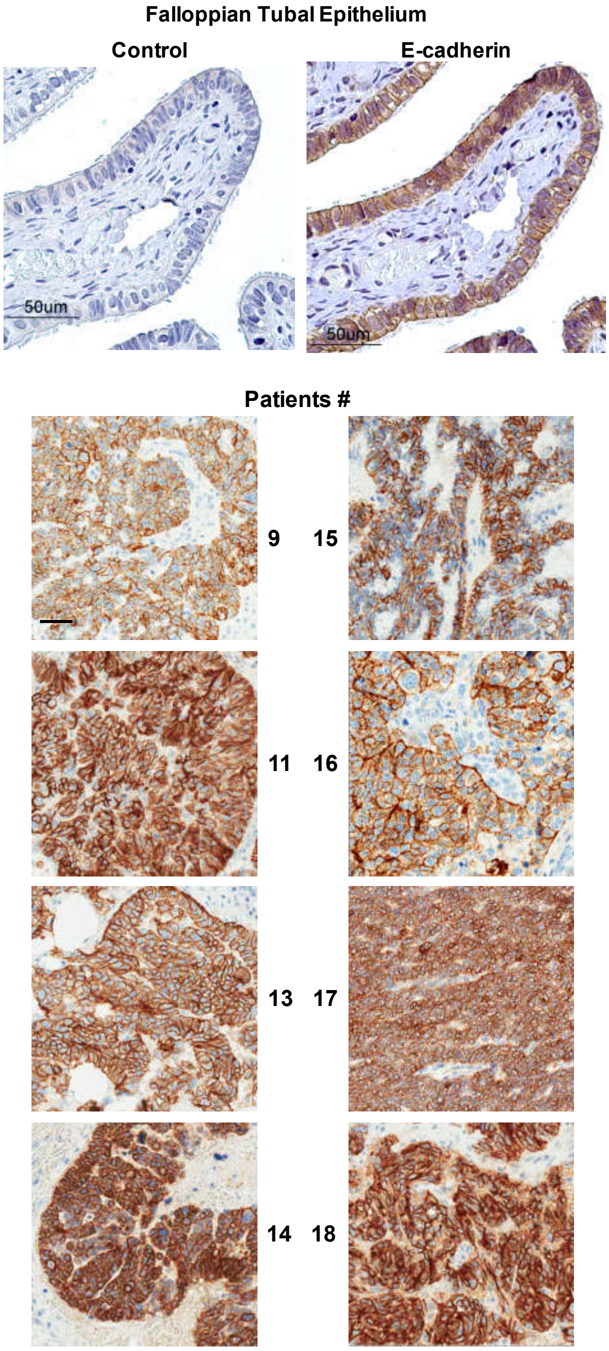

Supplementary Fig. 1

Supplementary Figure 1. Immunohistochemistry (IHC) with anti-E-cadherin on: upper panel, FFPE sections from fallopian tubal epithelium; and lower panel, eight FFPE samples of solid masses from sHG-SOC patients. Control, a section only processed with the secondary antibody. Bar, 50 µm.

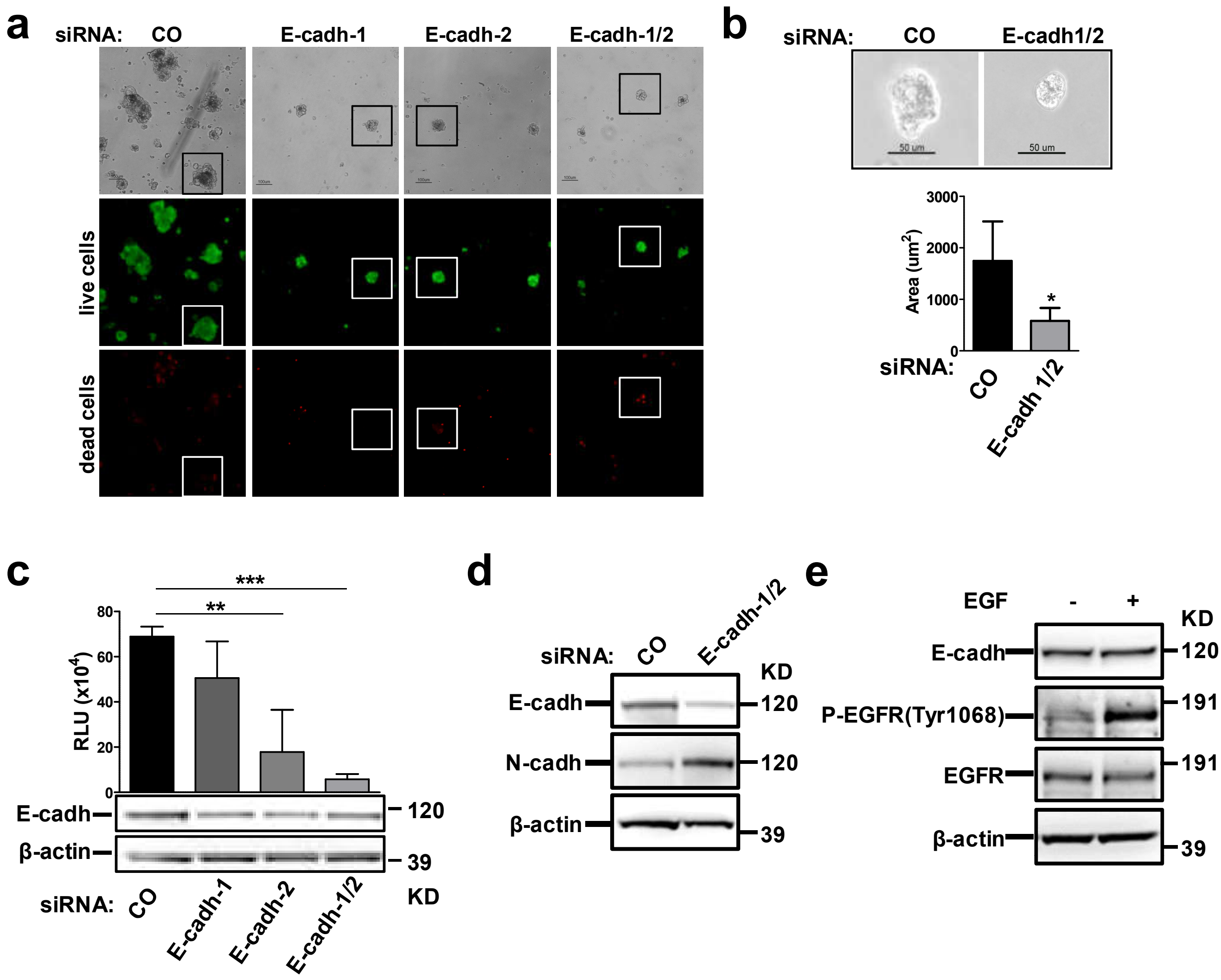

Supplementary Fig. 2

Supplementary Figure 2. a. Representative phase contrast images of OAW42 MCAs or fluorescent marked cells stained with LIVE/DEAD™ Viability/Cytotoxicity Kit (ThermoFisher Scientific); bar, 50 µm. The empty box highlights the image reported in Fig. 1f lower left panel. **b.** Upper panel: representative phase contrast images of MCAs of control (CO) and E-cadh (E-cadh-1/2) siRNA treated OAW42 cells grown in Matrigel® for 6 days. Lower panel: measurement of the area of OAW42 MCAs using ImageJ software. Asterisk indicates statistically significant values by Student's t test ($p<0.05$). **c.** OVCAR5 cells were transiently transfected with a control (CO) siRNA or with two E-cadherin siRNAs, separately (E-cadh-1, E-cadh-2) or pooled (E-cadh-1/2). Upper panel: cell viability assay performed on silenced OVCAR5; the number of cells was evaluated as mitochondrial activity with CellTiter-Glo® Luminescent Cell viability assay. Asterisks indicate statistically significant values by Student's t test ($p<0.01$). Lower panel: western blotting for evaluation of E-cadherin levels in OVCAR5 cells after 5 days of culture. **d.** Western blotting to evaluate E-cadherin silencing on experiments reported in Fig. 2c. OAW42 cells were transiently transfected with a control (CO) siRNA or with pooled E-cadherin siRNAs (E-cadh-1/2). Immunoblottings were performed with Abs against the proteins reported on the left. β-actin was used as a control for gel loading. **e.** Western blotting on lysates from OAW42 starved (-) for 24 hr and then stimulated with EGF 20 ng/ml (+) for 15 min. Immunoblottings were performed with Abs against the proteins reported on the left. β-actin was used as control for gel loading.

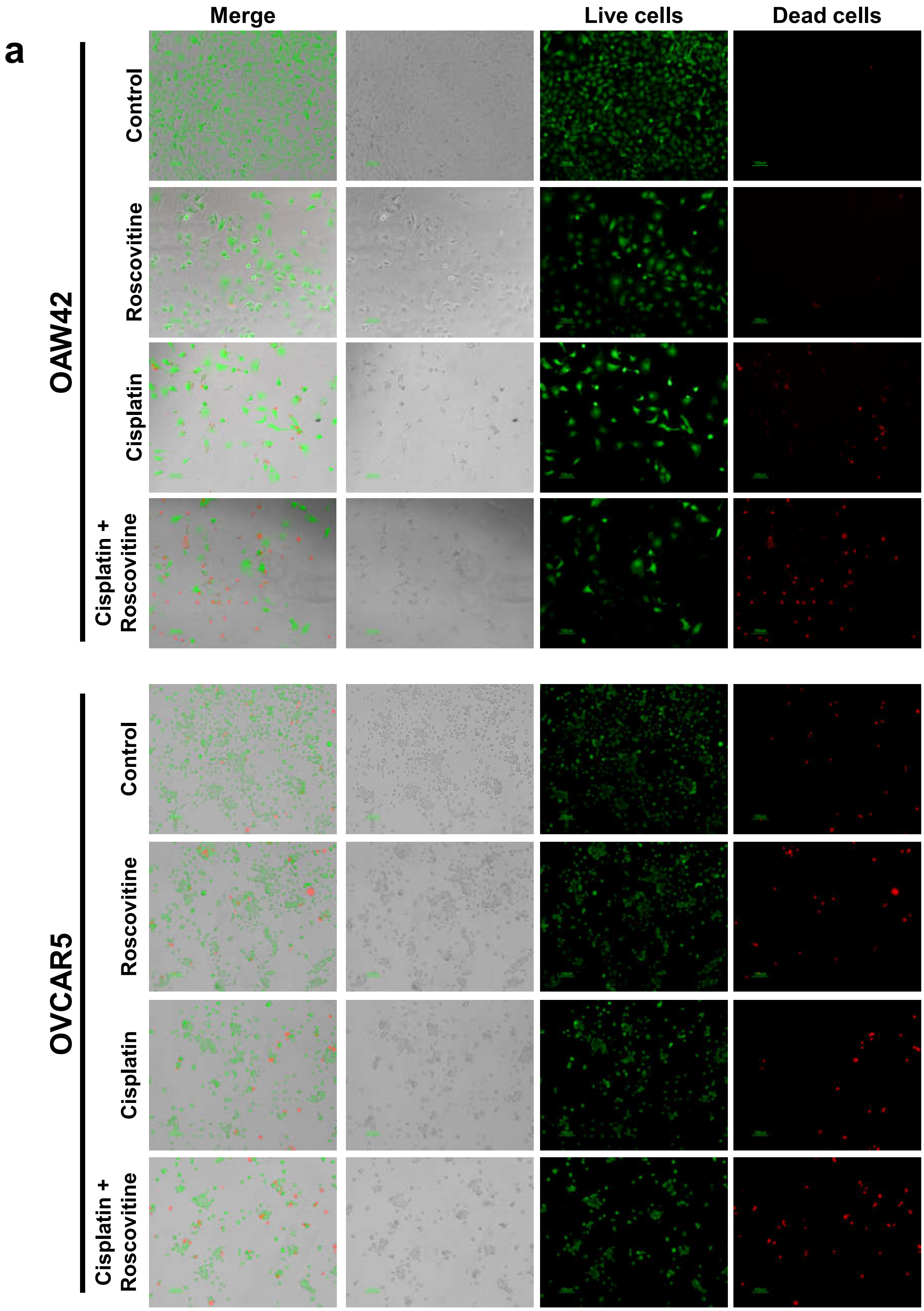

Supplementary Fig. 3

Supplementary Figure 3 . Representative phase contrast images or fluorescent marked OAW42 and OVCAR5 cells stained with LIVE/DEAD™ Viability/Cytotoxicity Kit (ThermoFisher Scientific); bar, 100 μm. Cells were treated with 10 μM roscovitine or cisplatin, 3 or 1,5 μM respectively, alone or in combination for up to 48 h.

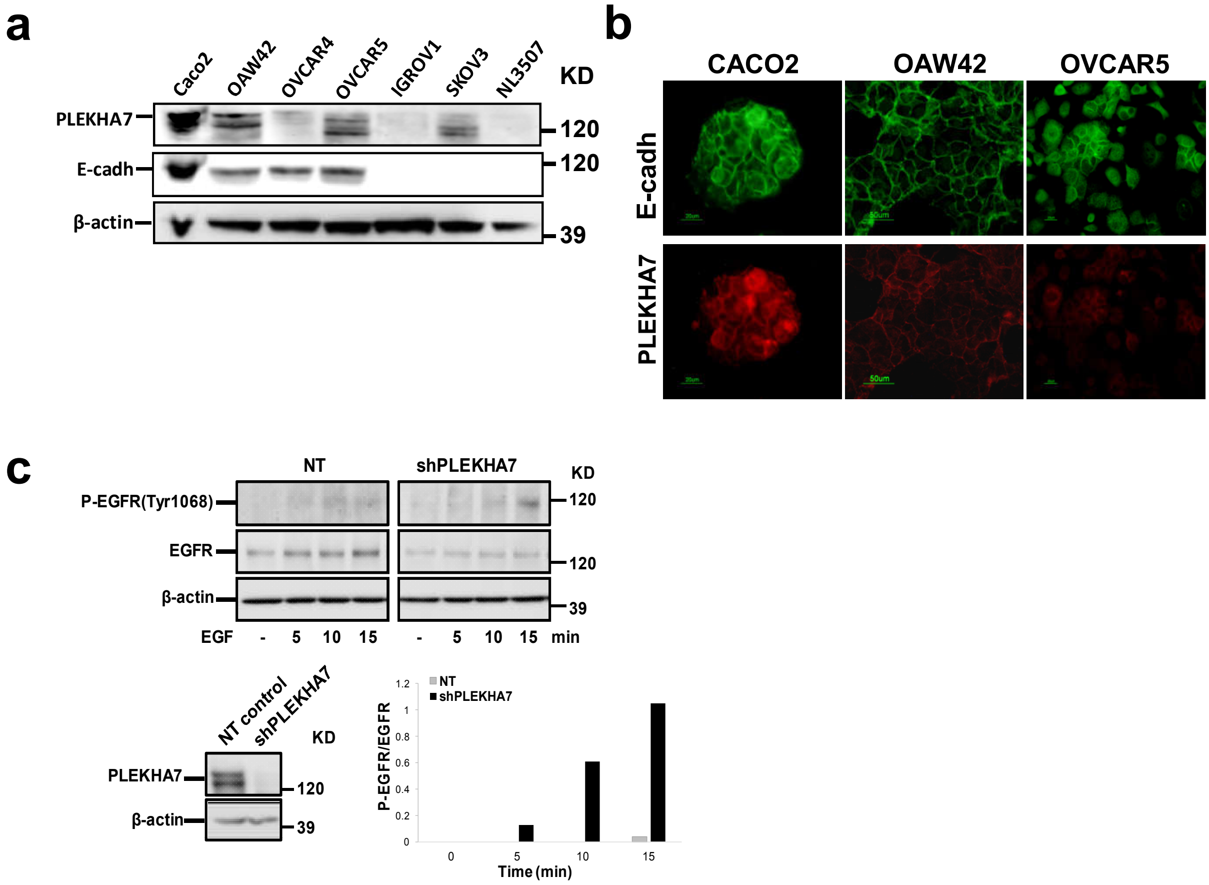

Supplementary Fig. 4

Supplementary Figure 4. a. Western blotting on total cell lysates from six EOC cell lines. Immunoblottings were performed with Abs against the proteins reported on the left. β-actin was used as control of gel loading. b. IF performed on fixed Caco2, OAW42, and OVCAR5 cells. Immunostaining was performed with anti-E-cadherin (cadh, green) and anti-PLEKHA7 (red) Abs. Caco2 was used as positive control of PLEKHA7 expression and localization. c. Upper panel: representative western blotting on lysates from Caco2 cells infected with a control (NT) shRNA or with PLEKHA7 shRNA (shPLEKHA7). Cells were starved (-) for 24 hr and then stimulated with EGF 20 ng/ml for 5, 10 and 15 min. Immunoblottings were performed with Abs against the proteins reported on the left. β-actin was used as control for gel loading. Lower left panel: western blotting with anti-PLEKHA7 Ab to evaluate PLEKHA7 silencing. Lower right panel: quantitative evaluation of P-EGFR on PLEKHA7 silenced cells of the experiment reported in the upper panel. The graph reports the ratio P-EGFR/EGFR.

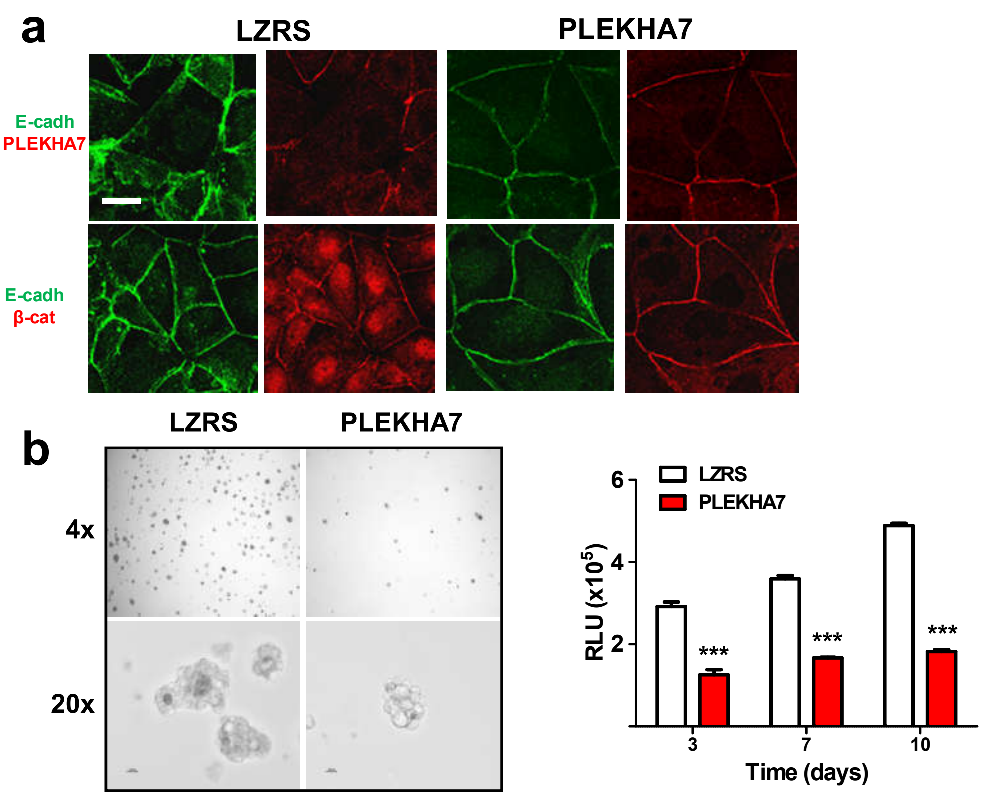

Supplementary Fig. 5

Supplementary Figure 5. a. Confocal IF performed on empty LZRS or LZRS-PLEKHA7 infected OAW42 cells immunostained with Abs reported on the left. Images of single Ab staining are shown. Bar, 20 µm. The panel reports the stacks reported in Fig. 5d as merge images. **b.** Left panel: representative phase contrast images of spheroids of LZRS or PLEKHA7 infected OAW42 cells grown in Algimatrix™ for 15 days. Morphological changes in spheroids shape and dimension were monitored using an inverted microscope with a 4X or 20X 0,75 NA PanFluor objective (Nikon). Right panel: cell viability assay performed on the same cells; the amount of cells was evaluated with the CellTiter-Glo® Luminescent Cell viability assay after the dissolution of the sponge. Asterisks indicate statistically significant values ($p \leq 0.0001$).
